# Vitamin K and Osteoporosis

**DOI:** 10.3390/nu12123625

**Published:** 2020-11-25

**Authors:** Maria Fusaro, Giuseppe Cianciolo, Maria Luisa Brandi, Serge Ferrari, Thomas L. Nickolas, Giovanni Tripepi, Mario Plebani, Martina Zaninotto, Giorgio Iervasi, Gaetano La Manna, Maurizio Gallieni, Roberto Vettor, Andrea Aghi, Lorenzo Gasperoni, Sandro Giannini, Stefania Sella, Angela M. Cheung

**Affiliations:** 1 National Research Council (CNR), Institute of Clinical Physiology (IFC), 56124 Pisa, Italy; dante.lucia11@gmail.com (M.F.); iervasi@ifc.cnr.it (G.I.); 2 Department of Medicine, University of Padova, 35128 Padova, Italy; roberto.vettor@unipd.it; 3 Department of Experimental Diagnostic and Specialty Medicine (DIMES), Nephrology, Dialysis and Renal Transplant Unit, S. Orsola Hospital, University of Bologna, 40138 Bologna, Italy; giuseppe.cianciolo@aosp.bo.it (G.C.); gaetano.lamanna@unibo.it (G.L.M.); lorenzo.gasperoni3@gmail.com (L.G.); 4 Department of Biomedical Experimental and Clinical Sciences “Mario Serio”, University of Florence, 50139 Florence, Italy; marialuisa.brandi@unifi.it; 5 Department of Medicine, Service of Bone Diseases, Faculty of Medicine and Geneva University Hospital, 1205 Geneva, Switzerland; serge.ferrari@unige.ch; 6 Department of Medicine, Division of Nephrology, Columbia University, New York, NY 10032, USA; tln2001@cumc.columbia.edu; 7 CNR-IFC, Clinical Epidemiology of Renal Diseases and Hypertension, Ospedali Riuniti, 89124 Reggio Calabria, Italy; gtripepi@ifc.cnr.it; 8 Laboratory Medicine Unit, Department of Medicine, University of Padua, 35128 Padua, Italy; mario.plebani@unipd.it (M.P.); martina.zaninotto@aopd.veneto.it (M.Z.); 9 Department of Biomedical and Clinical Sciences ‘Luigi Sacco’, Università di Milano, 20157 Milano, Italy; maurizio.gallieni@unimi.it; 10 Department of Medicine, Clinica Medica 1, University of Padua, 35128 Padua, Italy; andrea.aghi@gmail.com (A.A.); sandro.giannini@unipd.it (S.G.); stefania.sella@unipd.it (S.S.); 11 Department of Medicine, University Health Network, University of Toronto, 200 Elizabeth Street, Eaton North 7-221, Toronto, ON M5G 2C4, Canada

**Keywords:** vitamin K, vitamin K-dependent proteins, Osteocalcin, matrix Gla protein, vitamin K levels, vitamin K deficiency, bone fractures

## Abstract

Vitamin K acts as a coenzyme of carboxylase, catalyzing the carboxylation of several vitamin K dependent proteins. Beyond its well-known effects on blood coagulation, it also exerts relevant effects on bone and the vascular system. In this review, we point out the relevance of an adequate vitamin K intake to obtain sufficient levels of carboxylated (active form) vitamin K dependent proteins (such as Osteocalcin and matrix Gla protein) to prevent bone health. Another bone-related action of Vitamin K is being a ligand of the nuclear steroid and xenobiotic receptor (SXR). We also discuss the recommended intake, deficiency, and assessment of vitamin K. Furthermore, we review the few available studies that have as pre-specified outcome bone fractures, indicating that we need more clinical studies to confirm that vitamin K is a potential therapeutic agent for bone fractures.

## 1. Introduction

Nutrition represents one of the most relevant modifiable for osteoporosis prevention and bone health. [1]. Although impairment of bone remodeling is the cornerstone of the pathogenesis of primary osteoporosis, other related mechanisms have to be investigated. Intrinsic changes within the osteoblast and osteoclast lineage that may occur with ageing leading to an increased cellular senescence that is considered a hallmark of ageing may play a role [2,3].

Several studies have shown that nutrients and vitamins, including vitamin D, vitamin C, and recently vitamin K play an important role in the maintenance of optimal bone health, especially among older adults [4]. Recently, a growing interest has been directed to vitamin K, mainly involved in the blood coagulation pathway, since it maintains the activity of coagulation factors in the liver [5]. Epidemiological studies suggested that a vitamin K deficiency is associated with several diseases, including osteoporosis and vascular calcification [6].

## 2. Vitamin K Structure, Source, Cycle, Metabolism

Vitamin K is the term used to name a family of fat-soluble compounds, which although differ in origin and/or function, share a common 2-methyl-1,4-naphthoquinone ring, but differ in the (lipophilic) side chains linked at the 3-position. The three main forms are vitamin PK or phylloquinone (PK), vitamin K2 or menaquinones (MKn) and vitamin K3 or menadione. These three forms can be differentiated by the 3-position: PK has an isoprenoid side chain, whereas the MK form possesses a phytyl side chain and is characterized by a variable number of connected isoprenoid units (MKn), and menadione has no side chain and is a synthetic analogue [4].

The sources of vitamin K are different depending on the vitamers. PK can be found mainly in green leafy vegetables (e.g., kale), vegetables in the Brassica genus (e.g., Brussels sprouts, broccoli), fruits (e.g., avocado, kiwi, and green grapes), herbs (e.g., cilantro, parsley), and green and herbal teas. Other dietary sources are plant oils such as soybean, canola, and olive oils [7].

Fermented foods such as fermented butter or cheese, curdled cheese, egg yolk, and beef liver are sources of MKn. Natto, a traditional Japanese soybean-based food, produced by fermentation using *Bacillus subtilis*, is a source of Menaquinone-7 (MK-7) [7]. Regarding MKn produced by intestinal bacterial flora, MK-6, MK-7, and MK-8 are sinthetyzed respectively by *Eubacterium lentum*, Veillonella and Enterobacteria while MK-10 and MK-11 by Bacteroides. Differently MK-4, is converted from PK through a side chain removal/addition mechanism in specific tissues (pancreas, testes, and vessel wall) having menadione as intermediate molecule, but its synthetic form is added in animal food and is then metabolized by the liver into MK-4 [4,8].

Vitamin PK, MK-4, and MK-7 are the currently commercially available formulations. The synthetic form of vitamin PK, also known as phytonadione, is used to treat and/or prevent vitamin K deficiency, bleeding and reverse the consequences of an overdose of anticoagulant drugs [9]. Dietary vitamin K is absorbed in the small intestine, being fat-soluble, both forms of vitamin K need a normal pancreatic function and the presence of bile salts for their absorption and are transported in plasma by lipoproteins [10]. Plasma concentrations of PK in healthy fasting people are around of 0.5 nM, an order of magnitude remarkably lower than blood concentrations of other fat soluble vitamins (A, D, and E). Concentrations of MKs in plasma, such as MK-4, are very low or undetectable [11]. The low plasma concentrations of vitamin K are physiologically associated with tissue reserves. As vitamin K is stored in the body in a limited amount without a regular dietary intake, it would run out quickly. For this reason, the human body recycles vitamin K, compensating for its limited storage capacity [4].

Vitamin K acts as a cofactor for a single microsomal enzyme, namely carboxyglutamyl carboxylase (GGCX) which catalyzes the carboxylation (and thereby activation) of vitamin-K-dependent proteins (VKDPs) [12]. Vitamin-K-dependent carboxylase catalyzes the posttranslational γ-carboxylation of glutamic acid (Glu) residues, placed in vitamin K-dependent proteins (also known as Gla proteins), to γ-carboxylated glutamic acid (Gla) residues. Among the Gla protein family, 17 different members have been recongnized: S prothrombin, factor VII, factor IX, factor X, protein C, protein S and protein Z belonging to the coagulative cascade; (matrix Gla protein,(MGP), Osteocalcin (OC), growth arrest-specific protein 6 (Gas6), Gla-rich protein (GRP) playing a role in modulating bone and vascular mineralization; periostin and periostin-like factor, two proline-rich Gla proteins, and two transmembrane Gla proteins. Vitamin K was previously considered only an essential factor for blood coagulation [5].

Considering that some Gla-proteins are involved in bone metabolism and vascular health, it is possible that their reduced carboxylation may lead to bone metabolism impairment and/or an increase in vascular calcification [13]. Recently, it was found that vitamin k may also have regulatory functions in energy metabolism and a protective role against vascular calcification and age-related bone loss. The coenzymatic (active) form of vitamin K is hydroquinone (KH2), which is produced by a quinone reductase at the expense of NADPH. This reaction leads to the oxidation of KH2, to epoxide (KO). KH2 is restituted by KO reduction through two reductase activities: vitamin K epoxide reductase (VKOR) which first transforms KO into quinine and then vitamin K reductase reduces K quinone to the K hydroquinone (KH2). The activity of vitamin K reductase is needed to synthesize KH2, the active cofactor for the GGCX in the endoplasmic reticulum, as well as to reduce ‘new’ molecules of vitamin K that are introduced into the cycle. The vitamin K recycling reduces its dietary requirements ensuring its availability for the important coenzymatic function of carboxylation [14].

**Figure 1:**
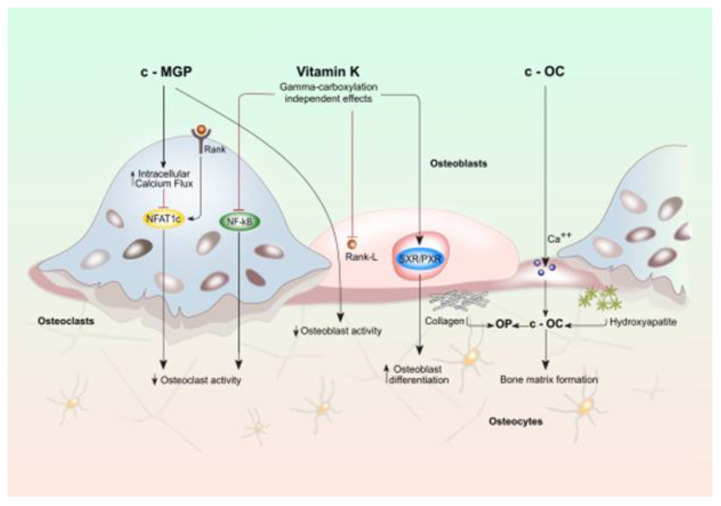
**Effects of vitamin K on activity of osteoblasts and osteoclasts. Bone cell function is modulated by both vitamin k dependent carboxylated proteins (c-MGP and c-OC) and vitamin K directly (gamma-carboxylation independent effects).** Vitamin K (VK) plays a considerable role in maintaining bone strength since it regulates bone remodeling by promoting the osteoblast-to-osteocyte transition and by limiting osteoclastogenesis. This effect would take place mainly through the VK-dependent activation of bone Gla-proteins: Osteocalcin (OC) and matrix Gla protein (MGP protein). OC secreted by osteoblasts, plays an essential role in the synthesis and regulation of bone matrix. The undercarboxylated OC, shows a reduced calcium and hydroxyapatite binding activity, while, the active carboxylated (cOC) form is mainly involved in bone mineralization as it allows the interaction between its calcium-binding Gla residues with the calcium ions of hydroxyapatite: the main mechanism that enables OC to contribute to the formation of hydroxyapatite crystals. Oc acts also as an inhibitor of bone mineralization thus regulating the rate of mineral maturation (see also the text for the details).

The involvement of vitamin K in bone metabolism does not occur exclusively by the γ-carboxylation reaction. MK4, binds to the nuclear receptor, steroid, and xenobiotic receptor (SXR) and its murine ortholog, pregnane X receptor (PXR), a nuclear receptor whose primary function is to regulate the expression of genes encoding enzymes involved in steroid metabolism and detoxification of xenobiotics and various drugs [15,16,17]. Vitamin PK is not able to activate SXR directly, suggesting vitamin PK potentially contribute to this mechanism of action after being converted into MK-4 [18] (Figure 1).

The presence of SXR has been demonstrated in the liver, intestine, and human osteoblastic cells. After binding with a ligand, SXR forms a complex with retinoid X receptor which in turn binds to an SXR responsive element on the target gene promoter that rules the transcription. MK-4, in an SXR-dependent way, plays a pivotal role in bone health by inducing expression of genes coding proteins such as matrilin-2 (Matn2), tsukushi (Tsk), and CD14 which are involved in bone remodeling [4,6,18] (Figure 1). Specifically, Tsk encodes a protein that has a collagen-accumulating effect, while Matn2 is a widely distributed extracellular matrix protein like collagen and CD14 regulates osteoblastogenesis and osteoclastogenesis by inducing differentiation of B cells [4,14,19]. The effect of vitamin K through SXR on bone collagen content may be important for bone quality. The material properties of bone, degree of mineralization, and microdamage accumulation are all influenced by collagen cross-link formation. In particular the type-I collagen fibers wired with crosslinks fibers form the framework that binds matrix proteins and mineral crystals. If the arrangement of collagen fibers is altered and the mineral crystal remains immature, these changes in material properties can cause an impairment of bone elasticity [20,21]. Moreover, SXR/PXR promotes bone formation and blunts bone resorption, suggesting that SXR/PXR may play a pivotal role in maintaining bone homeostasis [19].

The effect of VK on bone health and remodeling also involves MGP that promotes bone formation by upregulating Wnt/β-catenin signaling but also exerts an inhibitory effect on bone mineralization. In the late stage of osteoclast differentiation, MGP is highly expressed thus outlining a negative-feedback loop to make osteoclast formation under tight control (see also the text for the details).

Recent evidence suggests that VK regulates osteoblastogenesis and osteoclastogenesis through the nuclear factor κB (NF-κB) signal transduction pathway. NF-κB signaling exerts two functions: one the one hand it stimulates osteoclasts development and resorption while on the other it inhibits osteoblasts differentiation and activity. VK2 prevents NF-κB activation, in a γ-carboxylation-independent manner, leading to bone formation and reducing bone resorption. [22,23] (Figure 1).

## 3. Vitamin K and VKDPs

VK plays a considerable role in maintaining bone strength since it regulates bone remodeling by promoting the osteoblast-to-osteocyte transition and by limiting osteoclastogenesis. This effect would take place through the VK-dependent activation of the following bone proteins: Osteocalcin (OC), matrix Gla protein (MGP protein), Gla-rich protein, protein S, and growth arrest specific 6 protein (Gas6). While the role of OC and MGP in bone health and in cardiovascular health seems sufficiently detailed, the role of the other bone VKDPs appears less well-defined [9,24].

OC is the most plentiful non-collagenous protein in bone, mainly secreted by osteoblasts, with a smaller amount produced by chondrocytes. It plays an essential role in the synthesis and regulation of bone matrix. The carboxylation pathway, requiring vitamin K as a cofactor, is crucial for the transformation of OC from the undercarboxylated form into the fully functional carboxylated form. The plasma levels of OC are considered measures of bone formation [25] and there is a general consensus in considering it a marker of bone formation.

The undercarboxylated OC (ucOC), the inactive form, shows a reduced calcium and hydroxyapatite binding activity, while the active carboxylated (cOC) form is mainly involved in bone mineralization as it allows the interaction between its calcium-binding Gla residues with the calcium ions of hydroxyapatite. This property has been proposed as the main mechanism that enables OC to contribute to the formation of hydroxyapatite crystals [26]. The level of ucOC represents a more sensitive marker of vitamin K status in humans and therefore can detect subclinical vitamin K deficiency [27]. Indeed, a high ucOC level, considered a measure of low vitamin K levels and intake can be released during osteoclastic resorption [28]. Furthermore, the OC also acts as an inhibitor of bone mineralization thus regulating the rate of mineral maturation. This finding is reflected in the ability of OC to inhibit the precipitation of calcium salts from saturated solutions [29] and to prevent over-mineralization as observed in rodents treated chronically with warfarin [30]. Moreover, Ducy et al., in OC null mouse, showed an age-dependent increase in bone formation rate and bone mass [31].

Finally it has been hypothesized that the OC can also perform a mechanical function within the bone matrix since it binds hydroxyapatite and forms a complex with collagen through the matrix protein osteopontin, acting as a bridge between the matrix and mineral component of bone tissue [32,33,34].

The effect of VK on bone health and remodeling is not limited to OC but also involves MGP, another member of the Gla family. MGP is a 12-KDa gamma-carboxyglutamic acid-containing protein, synthesized by vascular smooth muscle cells, endothelial cells, osteoblasts, chondrocytes, and osteoclasts. MGP is one of the most potent endogenous inhibitors of vascular calcification in vivo. It directly prevents calcium phosphate precipitation through binding calcification crystals in blood vessels to form vesicles and apoptotic bodies, and inhibits trans-differentiation of vascular smooth muscles cells into an osteogenic phenotype by binding with BMP2 [35,36,37,38]. MGP plays a multifaceted effect in bone health since it not only promotes bone formation by upregulating Wnt/β-catenin signaling, but it also exerts an inhibitory effect on bone mineralization [39]. In addition, MGP inhibits osteoblast mineralization and affects bone mass by regulating the deposition of the bone matrix [40,41].

Notwithstanding the low MGP expression in mature osteoclast, its role in osteoclastogenesis is relevant. It is likely that extracellular MGP produced by other cells, such as osteoblasts and chondrocytes, may regulate the cross talk between osteoclasts and these cells.

Osteoclasts differentiate from cells of monocyte/macrophage lineage upon the sequential stimulation by two pivotal factors: the monocyte/macrophage colony-stimulating factor (M-CSF) and receptor activator of nuclear factor-kappa-B ligand (RANKL). RANKL selectively induces the nuclear factor of activated T cells, cytoplasmic 1 (NFATc1), the key transcriptional factor in osteoclastogenesis [41,42] whose nuclear translocation and activation [43,44] is regulated by the activation of calcium-calcineurin pathway [45]. Osteoclast differentiation and bone resorption are accelerated by MGP depletion and are suppressed by MGP overexpression. MGP downgrades several mechanisms involved in osteoclast differentiation and function; in particular, it attenuates the integrin-induced activation of Src/Rac1 canonical pathway and interferes with the Ca2 flux induction of NFATc1 activation [45,46]. Despite MGP expression is induced significantly by RANKL, it reduces osteoclast differentiation and formation. It has been hypothesized that in the late stage of osteoclast differentiation, MGP is highly expressed thus outlining a negative-feedback loop to make osteoclast formation under tight control [45]. Recently, these experimental data were confirmed by Evenoepel et al. who showed an association between poor vitamin K status, defined by dephosphorylated uncarboxylated MGP (dp-ucMGP) > 500 nmol/L, and an increased risk of incident fractures in 468 end-stage renal disease patients [47].

Posttranslational modification of MGP (i.e., phosphorylation of up to three serine residues and gammacarboxylation of up to five glutamate residues) may explain its different biological effects. While the role of carboxylation, which depends on Vitamin K, is better understood and determines MGP’s bioactivity as a calcification inhibitor, the function of phosphorylation is not yet clarified. Recent data suggest a role of phosphorylation in regulating MGP secretion into the extracellular milieu [48,49]. Such reactions do not proceed in parallel, so at least four different molecules can be found in circulation: (i) (dp-ucMGP; (ii) dephosphorylated carboxylated MGP (dp-cMGP); (iii) phosphorylated uncarboxylated MGP (p-ucMGP); (iv) phosphorylated carboxylated MGP (p-cMGP) according to the state of carboxylation and/or phosphorylation. The active form is both phosphorylated and carboxylated (p-cMGP) and its synthesis is stimulated by vitamin D [50].

## 4. Vitamin K Recommended Intake, Deficiency, and Assessment

The recommended daily intake (RDI) or adequate intake (AI) of vitamin K is aimed at ensuring normal blood coagulation [16]. There is some variability of these recommended target values across various organizations. The National Academy of Medicine in the US stated the AI of vitamin PK at 120 μg/day for adult men and 90 μg/day for adult women [51]. The World Health Organization and the Food and Agriculture Organization recommended dosages for vitamin PK at 65 μg/day for men and 55 μg/day for women, based on a calculated requirement of 1 μg/day/kg body weight [52]. Finally, the European Commission has established a recommended daily allowance (RDA) for vitamin K at 75 μg/day [53]. In 2012, the Italian LARN (Reference Assumption Levels for Nutrients and Energy), proposed by the Human Nutrition Italian Society (SINU) suggested an intake of vitamin K stratified for age (140 or 170 μg/day for 18–59 and >60 years old, respectively) [54].

However, the studies carried out so far have suggested that a relatively higher vitamin K intake is required for bone and vascular health. Since vitamin K is stored mainly in the liver where it is used for the maintenance of the normal coagulation balance, a greater amount is required for extrahepatic tissues [55,56]. A Tsugawa et al., analyzed 1183 healthy adolescents, elaborating a new method for estimating vitamin K intake by a logarithmic regression equation. Authors showed that bone metabolism requires more vitamin K than blood coagulation: 155–188 and 62–54 μg/day, respectively [57].

Binkley et al. showed that a vitamin K intake > 250 μg is required for γ-carboxylation of OC. These studies suggest that the effect of vitamin K deficiency is more prominent on bone rather than on blood clotting [58].

Moreover, since no toxicity data are available, a safe upper limit for vitamin PK has not been established. Recent studies have suggested that vitamin K2 may be more biologically active than vitamin PK; however, lacking sufficient data, there are no recommendations for intake values for vitamin K2 [9]. The assessment of VK plasma levels is difficult as well as conditioned by numerous factors such as its low circulating concentration, the non-polar nature of the molecule and the interference of lipids. Additional variables that may influence VK plasma levels are affected by diet, inflammation, and the coexistence of chronic disease [59].

Undercarboxylated Gla-proteins, OC and MGP, have proven to be more sensitive than prothrombin time in detecting subclinical vitamin K deficiency representing functional tests useful to estimate vitamin K blood levels indirectly. Vitamin K deficiency status prevents VKDPs from acquiring their carboxylated form. The gamma-carboxyglutamate (Gla) domain is responsible for the high affinity binding of calcium ions, thus allowing coagulation factors and OC and MGP to interact with negatively charged membrane phospholipids.

Evaluation of the clinical impact of vitamin K requires the measurement of serum vitamin K homologue levels or dietary vitamin K intake. Vitamin K homologues include phylloquinone (vitamin PK) and menaquinones such as MK-4 and MK-7. Since any uncarboxylated VKDP is released by the cells into the bloodstream as a function of specific tissue activity, serum levels of uncarboxylated VKDPs can be a useful alternative marker of tissue-specific vitamin K deficiency or insufficiency.

Unfortunately, although several studies demonstrated a negative association between serum undercarboxylated VKDP levels and vitamin K status or dietary vitamin K intake, the criteria for detecting vitamin K deficiency in tissues are not defined.

While mild vitamin K deficiency does not induce considerable changes in coagulation pathway, extra-hepatic VKDP carboxylation is most affected by subclinical deficient states [60].

## 5. Vitamin K as Potential Therapeutic Target for Bone Fractures

Low plasma concentrations of VK are associated with a high risk of bone fractures in both northern Europeans and Asians populations of both sexes [61,62,63].

Several experimental studies have adressed the issue of the role of Vitamin K in bone metabolism. Wu et al. showed that both PK and MK (MK-4 and MK-7) inhibitor osteoclast-mediated effects on bone resorption in a dose dependent manner [64]. Furthermore, Rangel et al. demonstrated increased compact bone mass, increased bone formation markers and decreased bone resorption markers in ovariectomized (OVX) mice supplemented with Vitamin K [65]. Also, the effect of coadministration of vitamin K2 and other antiosteoporotic drugs, such as Teriparatide [66] and bisphosphonates, has been investigated [21].

In their meta-analysis, Hao et al. showed a statistically significant inverse association between dietary vitamin PK intake and risk of fractures (highest vs. the lowest intake, Relative Risks = 0.78, 95% CI: 0.56–0.99). The authors did not find any significant association between low vitamin PK and BMD [67]. Recently, 374 postmenopausal women with osteoporosis were studied showing a lower serum vitamin PK in the group with fractures (prevalent fractures: 0.53 (0.41), no fractures: 0.65 (0.66) μg/L, *p* = 0.04) and independently associated with fracture risk [68]. Dp-uc MGP was detectable in 97 (75%) participants with serum vitamin PK of 0.26 (0.15) μg/L, whilst PIVKA-II was above the clinical threshold in only 3.8% [68].

To date, a limited number of RCTs evaluated the effects of PK and K2 supplementation on fracture risk showing a potential positive effect and few trials are ongoing [69,70,71,72,73,74,75,76] (Table 1). In a double blind, randomized, controlled study, 244 postmenopausal women received MK-7 (180 μg MK-7/day) capsules or placebo for 3 years to investigate its effect on vertebral fractures. MK-7 significantly decreased the loss in vertebral height of the lower thoracic region at the mid-site of the vertebrae after 2 and 3 years [72] (Table 1). An interventional study 241 osteoporotic patients were enrolled in a 24-month randomized open-label study: in the control group (without treatment; n = 121) and the vitamin K2–treated group (n = 120), which received 45 mg/day orally MK-4 (45 mg/day orally). They found a reduction in the vitamin K2-treated group of the incidence of bone fractures (p = 0.0273) lower than the control group [76].

Recently, Mott et al. [77] published an update of their previous meta-analysis [78], mainly in light of the results of a review and statistical analysis, made by Bolland and colleagues [79] relative to 33 RCTs which raised serious doubts regarding their integrity and validity but also into consideration that more RCTs have been published in the meantime. Most of trials included in the systematic review by Mott et al. were carried out in postmenopausal or osteoporotic patients. Among studies reporting clinical fracture, six trials used vitamin K2 (five with 45 mg MK-4, one with 360 μg MK-7) and three used vitamin PK (ranging from 200 μg to 10 mg). Clinical fractures were lower in those who underwent vitamin K treatment (2.24% vs. 3.06%), however when the analysis was limited to low risk of bias studies, the effect was smaller (2.34% vs. 3.01%). The review by Mott et al. pointed out a considerable problem with differing methods for reporting and the diagnosis of fractures. Some trials did not provide useful data to be processed in meta-analysis; furthermore, most of the studies were conducted in Japanese populations and postmenopausal women; thus, further research is required to draw conclusions for the efficacy of vitamin K in other populations.

Participant selection based on baseline characteristics is an important issue and concerns both the BMD level of the participant and the presence of vitamin K deficiency. The enrollment in a trial of patients with normal BMD at baseline may make it hard to show its effect on fractures even if vitamin K does protect those with low BMD at baseline from fractures. Unfortunately, vitamin K baseline values were not available in many trials; a protective effect of vitamin K is likely to be achieved only in patients who are vitamin K deficient.

Mott et al., in their meta-analysis, classified the ECKO Trial as low risk of bias. In that trial, 440 postmenopausal women with osteopenia were randomized to either 5 mg of oral vitamin PK or placebo daily for 2 years. After 2 years, the investigators found daily vitamin PK supplementation increased serum vitamin PK levels by 10-fold, and decreased the percentage of undercarboxylated OC and total OC levels. However, vitamin PK supplementation had no effect on BMD or bone resorption, but determined a lower incidence of clinical fractures (HR = 0.45, *p* = 0.04) [74]. These data were corroborated by Fusaro et al. who carried out an observational study evaluating the association between PK and MKn levels and vertebral fractures assessed by quantitative vertebral morphometry in 387 hemodialysis patients. Authors found that 55.3% of patients had vertebral fractures and PK deficiency was the strongest predictor of vertebral fractures. Moreover, OC levels were lower in patients with high prevalence of vertebral fractures [80]. These findings suggest that the effect of vitamin PK on bone may not be related to an effect on BMD or bone turnover, but perhaps to an improvement of bone quality (e.g., material properties such as collagen crosslinks).

In Japan, MK4 is a well-established drug for osteoporosis treatment since 1960 (with a dosage of 45 mg/day orally) given the results of interventional studies that have demonstrated reduction in bone fractures incidence and improvement BMD [81].

## 6. Conclusions

Several points of experimental evidence seem to outline that vitamin K play an important role for bone health. Low vitamin K intake, low circulating levels of vitamin K, high serum levels of ucOC, or low serum levels of total OC have been associated with an increased risk of bone fractures in observational and RCT studies. However, the results of clinical trials are not resolutive and still remains matter of discussion whether supplementation with vitamin PK or vitamin K2 (or both) reduces the risk of vertebral or nonvertebral fractures given limitations inherent in the design of the trials that assessed these outcomes. Vitamin D status should be taken into account in future studies on vitamin K effects on bone, as there might be an interaction between the two molecules. Treating osteoporotic patients with vitamin K might have the additional advantage of protecting arteries from vascular calcification through its action on MGP.

The efficacy of vitamin K on fractures and bone quality needs to be ascertained in future large trials drawn to overcome problems still unsolved after previous studies and with sufficient statistical power to detect true and clinically meaningful effects. More evidence is needed about the effects of vitamin K supplementation at physiological and pharmacological doses and what the required dose of vitamin K is to ensure bone and vascular health.

## Figures and Tables

**Table 1:** Published and ongoing randomized controlled trials on the relationship between vitamin K and bone fractures having fractures as pre-specified outcome. Abbreviations: PK and K2, vitamin PK and K2; MK4, menaquinone 4, MK7, menaquinone 7; RCT, randomized clinical trial; DB, double blind; ESRD, end-stage renal disease; HD, hemodialysis; PD, peritoneal dialysis; d, day; m, month; w, week; y, year; CAC, coronary artery calcification; AUs, Agatson units.

**Published Clinical Trials**				
**Study**	**Participants, n**	**Follow-Up**	**Interventions**	**Results**
Tanaka et al. 2017 [69]	Female, osteoporosis, >65 years, 1983	2 y	K2 (45 mg/d) and risedronate (2.5 mg/d or 17.5 mg/w) vs. risedronate alone	No difference in fracture incidence
Jiang et al. 2014 [70]	Postmenopausal, female, 213	1 y	MK4 (45 mg/d) vs. alfacalcidol 0.5 µg/d Co-intervention: calcium 500 mg/d	Lower fracture incidence in MK4 group
Kasukawa et al. 2014 [71]	Women with postmenopausal osteoporosis aged > 60 years, 101	1 y	K2 (45 mg/d) Co-intervention: risedronate (17.5 mg/w)	No difference in vertebral fracture incidence
Knapen et al. 2013 [72]	Healthy postmenopausal women, 244	3 y	MK7 (180 µg/d) vs. placebo	Lower vertebrae height loss in MK-7 group
Inoue et al. 2009 [73]	Postmenopausal, female, Osteoporosis, 4378	4 y	MK4 (45 mg/d) plus calcium L-aspartate (1.2 g/d) or dibasic calcium phosphate (3 g/d) plus) vs. calcium alone	No difference in fracture incidence
Cheung et al. 2008 [74]	Postmenopausal women with osteopenia and normal VitD, 440	4 y	PK (500 µg/d) vs. placebo	Less clinical fractures in PK group
Ishida et al. 2004 [75]	Postmenopausal, female, Osteoporosis, 396	2 y	Six goups: K2 (45 mcg/d), estrogen plus medroxyprogesterone, etidronate, alfacalcidiol, controls (no treatment)	Less vertebral fractures in K2, estrogen, etidronate vs. controls.
Shiraki et al. 2000 [76]	Osteoporosis, female, 241	2 y	K2 (45 mg/d) vs. placebo plus 150 mg/day elemental calcium vs. calcium alone.	Less fractures in K2 group
**Ongoing Clinical Trials**				
**Study**	**Participants**	**Study Design, Follow-Up**	**Intervention**	**End-Point**
NCT01528800 iPACK-HD	ESRD on HD, CAC score ≥ 30 AUs	Phase 2 RCT, DB, 12 m	PK (10 mg three times a week) vs. placebo	*Primary*: compliance *Secondary*: vertebral and lumbar fractures incidence, coronary artery calcification progression, CV events
NCT03871322 The Vitamin K2 and D3 intervention Trial in Children and Adolescents with the Low-energy Fractures	Age < 18 years, low-energy fracture, vitamin D serum level < 30 ng/ml	RCT, DB, 3 m	VitD3 (2000 IU/d) plus MK7 (90 µg/d) or MK7 alone vs. placebo	Primary: Time to fracture healing
NCT02976246 RenaKvit	HD or PD > 3 months	Phase 4 RCT, DB, 2 y	MK7 (360 mcg/d) vs. placebo	Primary: arterial stiffness assessed by pulse wave, BMD change in the distal radial bone Secondary: bone fracture incidence, thromboembolic events, biomarkers changes
